# Synergistic Effects of Secretory Phospholipase A_**2**_ from the Venom of *Agkistrodon piscivorus piscivorus* with Cancer Chemotherapeutic Agents

**DOI:** 10.1155/2013/565287

**Published:** 2012-12-27

**Authors:** Jennifer Nelson, Kristen Barlow, D. Olin Beck, Amanda Berbert, Nathan Eshenroder, Lyndee Francom, Mark Pruitt, Kina Thompson, Kyle Thompson, Brian Thurber, Celestine H.-Y. Yeung, Allan M. Judd, John D. Bell

**Affiliations:** Department of Physiology and Developmental Biology, Brigham Young University, Provo, UT 84602, USA

## Abstract

Healthy cells typically resist hydrolysis catalyzed by snake venom secretory phospholipase A_2_. However, during various forms of programmed cell death, they become vulnerable to attack by the enzyme. This observation raises the question of whether the specificity of the enzyme for dying cells could be used as a strategy to eliminate tumor cells that have been intoxicated but not directly killed by chemotherapeutic agents. This idea was tested with S49 lymphoma cells and a broad range of antineoplastic drugs: methotrexate, daunorubicin, actinomycin D, and paclitaxel. In each case, a substantial population of treated cells was still alive yet vulnerable to attack by the enzyme. Induction of cell death by these agents also perturbed the biophysical properties of the membrane as detected by merocyanine 540 and trimethylammonium-diphenylhexatriene. These results suggest that exposure of lymphoma cells to these drugs universally causes changes to the cell membrane that render it susceptible to enzymatic attack. The data also argue that the snake venom enzyme is not only capable of clearing cell corpses but can aid in the demise of tumor cells that have initiated but not yet completed the death process.

## 1. Introduction

During programmed cell death, the plasma membrane undergoes a series of quantifiable biophysical changes. These include a decrease in membrane order and an increase in interlipid spacing. The degree of change in these membrane properties during apoptosis can be quantified using trimethylammonium diphenylhexatriene (TMA-DPH) and merocyanine 540 (MC540). This “softening” of the membrane has a facilitative effect on the activity of AppD49 snake venom secretory phospholipase A_2_ (sPLA_2_) causing the enzyme to readily hydrolyze membranes of damaged and dying cells while leaving membranes of healthy cells virtually untouched. The connection between cell health and phospholipase activity suggests the intriguing hypothesis that cancer cells treated with antineoplastic agents would become vulnerable to attack by the enzyme resulting in accelerated demise and clearance of these cells.

This study uses four chemotherapeutic agents that initiate apoptosis by different mechanisms (paclitaxel: microtubule inhibitor, methotrexate: inhibitor of thymine synthesis, actinomycin D: transcription inhibitor, and daunorubicin: DNA replication and transcription inhibitor) to answer two critical questions relevant to this hypothesis. First, do cells treated by each type of chemotherapeutic agent become susceptible to hydrolysis by sPLA_2_? Second, regardless of the induction mechanism, do cellular membranes exhibit the same changes in biophysical membrane properties as the apoptotic program proceeds?

## 2. Materials and Methods

### 2.1. Reagents

The monomeric aspartate-49 phospholipase A_2_ from the venom of *Agkistrodon piscivorus piscivorus* was isolated according to the procedure used by Maraganore et al. [1]. Ionomycin was purchased from Calbiochem (La Jolla, CA, USA). The fluorescent probes TMA-DPH, MC540, and propidium iodide were acquired from Invitrogen (Carlsbad, CA, USA). Methotrexate, daunorubicin, paclitaxel, and actinomycin D were obtained from Sigma-Aldrich (St. Louis, MO, USA). Other reagents were purchased from standard sources. Ionomycin, MC540, paclitaxel, and actinomycin D were dissolved in dimethylsulfoxide (DMSO); TMA-DPH in dimethylformamide; methotrexate in slightly basic modified balanced salt solution (134 mM NaCl, 6.2 mM KCl, 1.6 mM CaCl_2_, 1.2 nM MgCl_2_, 18.0 mM Hepes, 13.6 mM glucose, pH 7.4, MBSS); daunorubicin in water.

### 2.2. Cell Culture and Experimental Protocol

Cultures of S49 mouse lymphoma cells were grown in Dulbecco's Modified Eagle Medium at 37°C in humidified air containing 10% CO_2_ and prepared for experiments as described [2]. For spectral and kinetic spectrofluorometer experiments (FluoroMax 3, Horiba Jobin-Yvon, Edison, NJ, or PC-1, ISS, Champaign, IL, USA), an aliquot of cells (usually between 0.4×10^6^ and 3.0×10^6^ cells/mL in MBSS) was transferred to a quartz fluorometer sample cell and allowed 5 min to equilibrate. Spectral bandpass varied between 4 and 16 nm depending on the intensity of the probe and instrument sensitivity. Temperature and sample homogeneity were maintained as described previously [3]. All experiments and incubations were performed at 37°C. 

### 2.3. Membrane Hydrolysis

Membrane hydrolysis due to the action of sPLA_2_ was assayed by measuring the rate of propidium iodide (37 mM final) uptake by cells as described previously [2, 3]. Use of the propidium iodide assay as an indication of the number of cells hydrolyzed was validated using an intestinal fatty acid binding protein as explained [2]. After beginning data acquisition to obtain a measure of background signal due to scattered light, propidium iodide was added to the cells and allowed sufficient time to equilibrate. Phospholipase A_2_ was then added (70 nM final), and data acquisition continued until the intensity of propidium iodide fluorescence was stable. The detergent Triton X-100 was then added to permeabilize any remaining cells at the conclusion of the experiment. Data were analyzed as shown previously [3] and the percentage of cells in the three categories: “already dead;” “alive, but susceptible to sPLA_2_;” “resistant to sPLA_2_” was calculated.

### 2.4. Probes of Membrane Structure

Merocyanine 540 (170 nM final) data were acquired as emission spectra (excitation, 540 nm; emission, 550–700 nm). Spectra were obtained with cell samples before the addition of probe, 5 min after addition, and 10 min after addition of ionomycin (300 nM). The third spectrum after ionomycin treatment was used to establish the maximum response of 100% of the cells as described previously [3, 4]. Spectra were analyzed by subtracting the initial background spectrum from the other two and integrating the intensity from 565 to 615 nm. The integrated intensity of the second spectrum was then expressed as a fraction of that of the third.

Measurements of TMA-DPH steady-state fluorescence anisotropy (excitation = 350 nm; emission = 452 nm, 16 nm bandpass in both cases) were made in the PC-1 fluorometer, with the fluorometer in the “L” configuration and Glan-Thompson polarizers first both in the vertical position (parallel) and repeated with the excitation polarizer in the vertical position and the emission polarizer in the horizontal position (perpendicular). Data were obtained before the addition of the probe to measure background cell fluorescence and after 10 min equilibration with TMA-DPH (240 nM final). TMA-DPH fluorescence was analyzed by calculating the anisotropy (*r*):

(1) r=Ivv−GIvhIvv+2GIvh,

where *I*_*vv*_ and *I*_*vh*_ are the intensities with parallel and perpendicular polarizer configurations (after subtracting background light), respectively. *G* is the correction factor for transmission efficiency of the emission monochromator, calculated as follows:

(2) G=IhvIhh.

To control the effect of solvent and other factors, control experiments with just the solvent and all other variables intact were run in tandem for all procedures. 

## 3. Results

### 3.1. Susceptibility to sPLA_2_

**Figure 1:**
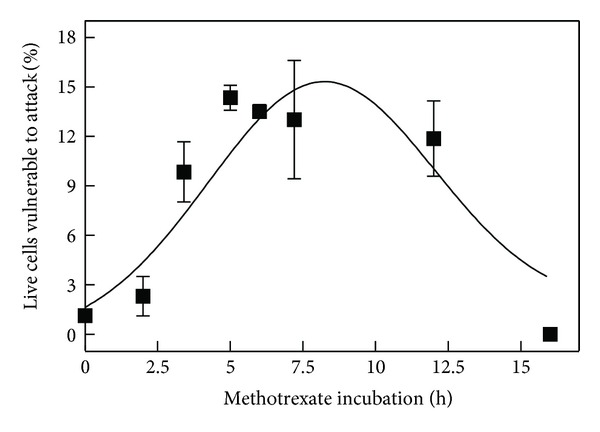
Evolution of “alive and susceptible” cell population during methotrexate-induced cell death. The percentage of the total cell population that was alive and susceptible to sPLA_2_ was assayed using propidium iodide at 1 h intervals during the apoptotic process (*n* = 3–5 for each methotrexate time point, *n* = 36 for control) as explained in Section 2.

**Figure 2:**
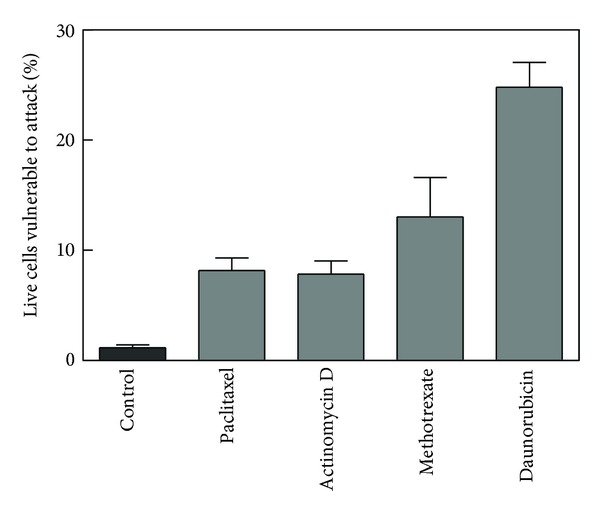
Comparison of maximum percentage of observed “alive and susceptible” population for cells treated with paclitaxel, actinomycin D, methotrexate, and daunorubicin. Data were analyzed by one-way analysis of variance followed by a Dunnett's posttest comparing methotrexate (*n* = 7), actinomycin D (*n* = 9), paclitaxel (*n* = 3), and daunorubicin (*n* = 4) to control (*n* = 21). The overall effect of treatment was significant (*P* < 0.0001) with all treatments differing individually from the control (*P* < 0.05).

The time needed to induce apoptosis in 100% of S49 cells varied from agent to agent (200 nM paclitaxel, 48 h; 32 nM actinomycin D, 24 h; 10 *μ*M methotrexate, 16 h; 20 *μ*M daunorubicin, 12 h). With each agent, cells became vulnerable to attack by sPLA_2_ during the process of cell death.  Figure 1 shows the rise and fall of the alive and susceptible population for cells that have been treated with methotrexate. Between 5 and 10 h, the proportion of the alive and susceptible population reached a maximum of 13% and then decreased as the cells succumbed completely to the apoptotic process. Results for the other three agents exhibited a similar time-dependent pattern (data not shown).  Figure 2 displays the maximum percentage of alive and susceptible cells for each of the four treatments. For paclitaxel and actinomycin D, the maximum occurred at 18 h. For daunorubicin, it was 7 h.

### 3.2. Physical Changes

**Figure 3:**
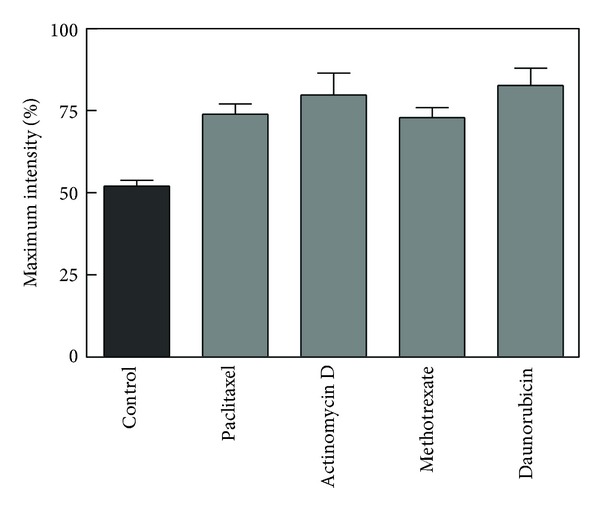
Effect of chemotherapeutic agents on MC540 fluorescence intensity. Cells were incubated with control vehicle or each agent and then harvested at the time when the alive and susceptible population reached a maximum. MC540 was added and assayed as described in Section 2. Data were analyzed by one-way analysis of variance followed by a Dunnett's post test comparing methotrexate (*n* = 3), actinomycin D (*n* = 6), paclitaxel (*n* = 3), and daunorubicin (*n* = 8) to control (*n* = 54). The overall effect of treatment was significant (*P* < 0.0001) with all treatments differing individually from the control (*P* < 0.05).

Figure 3 illustrates the change in MC540 fluorescence that corresponded in each case to the emergence of the largest alive and susceptible population. For each agent, the fluorescence intensity increased beyond that observed in control samples. However, in no case did it reach 100%, presumably since the entire population of cells was never uniformly synchronized in the program of cell death. The increased MC540 fluorescence intensity is caused by amplified binding of the probe to the cellular membrane, which is reflective of greater interlipid spacing [3, 5, 6].

**Figure 4:**
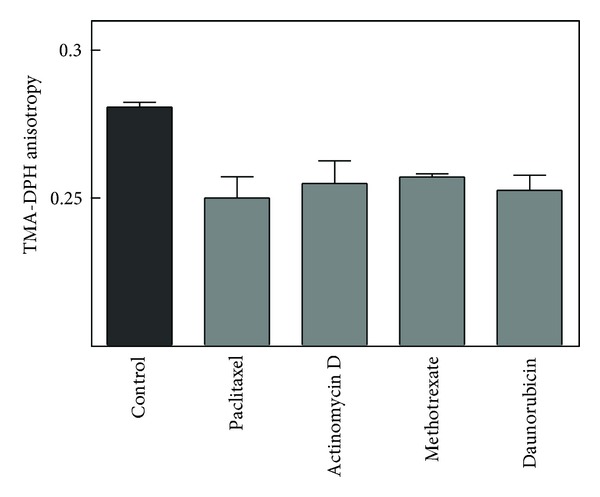
Effect of chemotherapeutic agents on TMA-DPH anisotropy. Cells were incubated with control vehicle or each agent and then harvested at the time when alive and susceptible population reached a maximum. TMA-DPH was added and anisotropy was assayed as described in Section 2. Data were analyzed by one-way analysis of variance followed by a Dunnett's posttest comparing methotrexate (*n* = 3), actinomycin D (*n* = 6), paclitaxel (*n* = 3), and daunorubicin (*n* = 3) to control (*n* = 10). The overall effect of treatment was significant (*P* = 0.0006) with all treatments differing individually from the control (*P* < 0.05).

In addition, the anisotropy of TMA-DPH was used to detect changes in cellular membrane lipid order. As shown in Figure 4, each drug caused a significant drop in anisotropy. This decrement in anisotropy is generally interpreted as an indication that the membrane had become less ordered or more fluid. 

## 4. Discussion

Prior investigations by our lab using calcium ionophore, glucocorticoid, and thapsigargin have successfully identified several biophysical changes that occur in the cellular membrane during apoptosis [3, 4, 7–9]. The results of this current study confirm that these changes are a generalizable feature of programmed cell death that occur regardless of the type of apoptotic inducer. Methotrexate, paclitaxel, actinomycin D, and daunorubicin induce cell death by disparate means; however, in each case, the interlipid spacing and fluidity of the membrane increased compared to the control. The idea is that these physical changes improve the likelihood of vertical movements of phospholipids in the membrane sufficient for individual phospholipids to enter the active site of adsorbed enzyme [3, 4, 7, 10–12]. For example, a recent study on thapsigargin-treated cells estimated that the chance of such lipid protrusions from the membrane increases approximately 100 fold when TMA-DPH anisotropy decreases to levels comparable to those displayed in Figure 4 [7]. In each case, the changed biophysical state of the membrane appeared to render it vulnerable to attack by snake venom sPLA_2_ as evidenced by the emergence of a significant population of cells that were alive and susceptible to hydrolysis.

This ability of snake venom sPLA_2_ to hydrolyze the membranes of cells dying by administration of antineoplastic agents suggests that it could potentially act as an adjuvant in facilitating the demise of cancer cells. There are two ways by which it could aid in the process. First, the enzyme can directly hydrolyze and permeabilize cells that have initiated apoptosis due to treatment with a chemotherapeutic agent. The fact that many of the cells hydrolyzed were still alive suggested that one could develop a strategy in which the dose of both antineoplastic agent and sPLA_2_ would be calibrated to maximize the number of cells that remain alive but are vulnerable to enzymatic destruction. In this way, untoward effects of the antineoplastic could perhaps be mitigated. Even if the combined use of enzyme and agent does not change the total number of cells eliminated during the chemotherapy, the data of this study suggest that the clearance of tumor cells could be accelerated, thus allowing the length of the treatment course to be reduced.

The second beneficial effect of combining sPLA_2_ with traditional chemotherapy is that one of the hydrolysis products released (lysophospholipid) can serve as a “find me” signal for macrophages [13]. This signal recruits macrophages to the vicinity of the cells experiencing hydrolysis. Those cells that additionally express phosphatidylserine on their surface are then eliminated by the macrophage. As shown previously, such expression of phosphatidylserine is a common feature of cells exposed to antineoplastic drugs [14–17]. The caveat to that idea is that the hydrolysis products are also proinflammatory, and failure of the macrophage system to adequately respond could result in unwanted stimulation of the immune system. 

This study is not the first to propose use of snake venom as an element of a chemotherapeutic regiment. For example, venom from *Hydrophis spiralis* has been shown to improve Ehrlich ascites carcinoma symptoms in mice treated additionally with 5-fluorouracil [18]. In addition, mammary cancer tumor load was reduced significantly and life span prolonged in mice treated with the venom of *Cerastes cerastes* [19]. Our study differs in using a purified component of the venom, that is, sPLA_2_ and proposes a mechanism to explain the enhanced susceptibility of cells to the enzyme during chemotherapy. Alternatively, endogenous sPLA_2_ has been associated with cancer in a variety of other contexts including the etiology of certain tumors [20–23], exacerbation of tumor proliferation [24], as a biomarker for some cancers [24–26], and as a means for delivery of liposome-encased drugs [27–29].

## 5. Conclusions

We have demonstrated here that lymphoma cells treated with diverse chemotherapeutic agents become vulnerable to enzymatic attack by sPLA_2_ prior to death. This effect therefore appears to be a general phenomenon of apoptotic cells [3, 4, 30, 31] and could potentially be exploited in designing chemotherapeutic strategies. The results also substantiate the concept that susceptibility to the enzyme relates to physical properties of the cell membrane such as those detected by MC540 and TMA-DPH. Future studies are needed to explore the range of applicability of these observations to various tumor types and to compare them to the behavior of the normal tissues from which the tumors originate. Preliminary results from our work on normal human lymphocytes suggest the possibility that some tumor lines may be substantially more susceptible to the action of snake venom sPLA_2_ than their normal counterparts [8].
